# Mr. Jones, in Answer to Mr. Hume

**Published:** 1810-07

**Authors:** Thomas Jones

**Affiliations:** Hickham, Lancashire


### *To the Editors of the Medical and Phyſical Journal.*

Gentlemen,

YOUR Correspondent, Mr. Hume, is perfectly correct when he observes that there is some "omission in the report" of my case of death by poison; it ought to have been written as it was originally in my Notes, thus· "Three grains of the powder with nine grains of kali were boiled, &c." The green colour produced by the second experiment, proves that the alkali was used. It was employed also in the first, in the above-mentioned proportion; and the junction of the two fluids produced a *turbid mixture* or compound of a sky blue colour; which, to the best of my recollection, deposited a precipitate on standing. I very much regret that this most material omission should have made the account so confused. That similar omissions of words, though not equally important, are occasionally made by others, will be evident to Mr. Hume if he will peruse the second paragraph of his own letter. This, however, is no justification of my own carelessness. The above, I conceive to be a sufficient answer to the four first queries. In answer to the fifth, I beg leave to observe, that with respect to the degree of heat to be employed, I do not feel myself competent to give a positive opinion; but a *red heat* during the space of ten minutes has been recommended, in making this experiment, by an eminent physician and chemist, vide Dr. Bostock's observations vations on detecting arsenic, Edin. Med. and Surg. Journ. April, 1809, where the following objection to the employment of dry charcoal is also to be found.

"When the dry charcoal is employed, the powder is in danger of escaping from between the plates, unless they be very closely pressed together; and when this is the case, a considerable part of it will be found unconsumed after they have been heated."

Every one must agree with Mr. Hume in the propriety of paying attention, in such cases, to what is evacuated from the stomach, when an opportunity is afforded: but, upon this occasion, the patient had not any medical attendant during her illness; and when we were called to inspect the body, two days after her decease, every utensil had been cleansed. The quantity of the white powder, and the facility with which I could obtain it, made me less inquisitive on that head; not having any doubt in my own mind but that I should be able, by analysing it, satisfactorily to prove that it was the cause of her death.

I have not, at present, an opportunity of repeating any of the experiments, nor of performing any of those recommended by your Correspondent. If, however, Mr. Hume is yet of opinion that the experiments were not sufficiently decisive, and imagines that it is possible to detect the arsenic, I will with pleasure give him an opportunity, by sending him, if he wishes it, when I return to Howden, the remaining five grains in my possession, with liberty to use it in any way he thinks most conducive to the promotion of science.

Before I conclude, I beg leave to thank him for the candour he has shewn in making his remarks; and which forms a striking contrast to the flippancy adopted by some of your Correspondents, and which, no doubt, has frequently prevented young practitioners, like myself, from contributing their useful mite when an opportunity presented. I am, &c.

THOMAS JONES.

*Hickham, Lancashire, June 13, 1810.*

